# Greater Risk Taking in Cosmetic Surgery Acceptance and History: An Experimental and Computational Study

**DOI:** 10.1007/s00266-024-03910-9

**Published:** 2024-03-21

**Authors:** Paul Mark Jenkinson, Elena Panagiotopoulou, Athanasios Koukoutsakis, Aikaterini Fotopoulou

**Affiliations:** 1 Research Department of Clinical, Educational and Health Psychology, University College London, Gower Street, London WC1E 6BT, UK; 2 Education and Training Division, Anna Freud, London, UK

**Keywords:** Cosmetic surgery, Attitudes, Acceptance, Risk taking, Computational

## Abstract

Cosmetic surgery is ever more affordable and accessible, but carries physical and psychological risks. Yet, no study to date has directly examined risk-taking behaviour under controlled conditions, beyond self-report and in relation to cosmetic surgery attitudes. We used the Balloon Analogue Risk Task and advanced computational modelling to measure decision-making behaviour and identify the latent parameters driving behaviour associated with cosmetic surgery attitudes in women with no cosmetic surgery history (*N* = 265) and a subsample of women with a cosmetic surgery history (*N* = 24). Risk taking was higher in women with greater acceptance and history of cosmetic surgery. Computational modelling revealed increased risk taking in women with greater acceptance of cosmetic surgery when decisions were made with greater knowledge of loss (*risk*) and not when the likelihood of loss was unknown (*uncertainty*). When women with greater acceptance of cosmetic surgery made decisions, they also placed less emphasis on possible losses (reduced *loss aversion*). Our findings suggest that women seeking cosmetic procedures may be less sensitive to losses and thus make more risky decisions. Greater emphasis should be placed on communicating potential losses rather than just the associated risks to women considering cosmetic procedures.

*No Level Assigned* This journal requires that authors assign a level of evidence to each submission to which Evidence-Based Medicine rankings are applicable. This excludes Review Articles, Book Reviews, and manuscripts that concern Basic Science, Animal Studies, Cadaver Studies, and Experimental Studies. For a full description of these Evidence-Based Medicine ratings, please refer to the Table of Contents or the online Instructions to Authors www.springer.com/00266.

## Introduction

The ability to modify one’s body is ever-more affordable and accessible for people in developed and developing countries. Cosmetic procedures comprise both surgical interventions (cosmetic *surgery*, i.e. operations that involve cutting beneath the skin to change the body’s appearance for primarily aesthetic rather than medical goals) and a range of nonsurgical interventions (cosmetic *treatments*: e.g. injectables such as botulinum toxin (Botox) and hyaluronic acid, laser skin treatments, and cryolipolysis (fat freezing)). Over 10 million cosmetic surgeries and 14 million nonsurgical cosmetic treatments were performed worldwide in 2020 [1]. Importantly, cosmetic surgery (which is the primary focus of the current work) carries potential risks, including physical complications (e.g. scarring, bacterial infection, and bleeding) and negative psychological (e.g. anxiety, disappointment, and depression) [2] and social outcomes (e.g. negative evaluation and stigmatisation) [3]. Despite a substantial rise in the number of people undergoing surgical and nonsurgical cosmetic procedures, the factors that influence peoples’ decisions, including their propensity towards risk, remain poorly understood.

Existing research in this field has mostly focused on the psychosocial and cultural factors that influence the likelihood of undergoing cosmetic surgery [see 4−6 for reviews], by examining attitudes and drives towards cosmetic surgery using questionnaires, interviews, or focus groups [2, 7−11]. Notably, Park and Cho [10] surveyed 298 undergraduates to examine which psychological and sociocultural factors explain the intention to receive cosmetic surgery in the future. They found that the perceived risk of cosmetic surgery was an important (significant) predictor of attitudes towards cosmetic surgery. However, no study to date has verified this association using experimental methods that allow risk-taking *behaviour* to be directly observed. Although self-report questionnaires and qualitative methods provide valuable insights into the conscious attitudes and self-reported behaviour associated with cosmetic surgery, experimental studies can provide further insights by examining people’s nonverbal behaviour under controlled laboratory conditions. Applying such methods to cosmetic surgery research stands to provide key insights about the psychological mechanisms that determine individual differences regarding risk and, particularly, differences in how people make decisions under risk.

Experimental, value-based decision-making tasks can quantify how people take decisions when faced with different options that have different perceived benefits and costs, including risk. People integrate such benefits and costs into a subjective value function according to their preferences, and make decisions accordingly [12]. These tasks can thereby quantify the relative influence of different benefits and costs on subjective value, and computational modelling of the observed choices can reveal some of the hidden drivers (latent parameters) underlying observable behaviour [see 13 for an introduction to these ideas]. For example, value-based decision-making tasks have revealed that although people normally prefer larger over smaller rewards, this situation changes when rewards are associated with costs, such as delays or risks [14]. The risks associated with cosmetic surgery may lead certain individuals to ‘discount’ the perceived rewards of cosmetic surgery, while other individuals may disregard such risks in favour of the perceived rewards associated with acquiring their chosen body appearance. However, no study to date has applied such methods to examine the relationship between attitudes and decisions about cosmetic surgery and risk-taking behaviours. This was the main aim of the current research.

We used the Balloon Analogue Risk Task [BART; 15], a widely accepted measure of risk-taking behaviour, during which participants must ‘pump’ a virtual balloon in order to ‘win’ money, but with every pump they also risk reaching a set but unknown ‘burst’ point in which all money from that trial is lost. Performance on the BART is related to real-world risk behaviours, including smoking [16], alcohol and substance use [17], gambling [18], risk-related sexual behaviour [19], and to personality traits including sensation seeking and impulsivity [20]. While the BART has some known limitations, particularly conflating decision-making under uncertainty (when outcome probabilities are *not* known) and risk (when outcome probabilities *are known*) [21, 22], as well as risk aversion (i.e. sensitivity to the value of reward) rather than loss aversion (i.e. sensitivity to negative outcomes; see Table 2 for further definitions), we have recently developed a dual, computational modelling approach that is capable of disambiguating between both sets of variables [23].

Using this approach here, we conducted the first experimental and computational study to assess risk taking in relation to cosmetic surgery attitudes and acceptance in a large sample of women without a history of cosmetic procedures, as well as in a second, independent but smaller sample of women who had undergone cosmetic procedures. We controlled for key characteristics such as body mass index [BMI; 24, 25], and related variables, including body image concerns, disordered eating, depression and anxiety. Based on previous self-report studies identifying risk-taking tendency as a factor that explains the intention to receive cosmetic surgery [10], we expected that greater acceptance of cosmetic surgery, or having undergone a cosmetic procedure, would predict a propensity to take greater risk during the BART. In further computational analyses we predicted that (after controlling for other variables) acceptance of cosmetic surgery would be associated with greater risk-taking behaviour during ‘risk’, but not ‘uncertainty’ trials and would be related to risk—rather than loss aversion.

## Materials and Methods

### Participants

We recruited 302 women to a laboratory-based study conducted at University College London. Participants responded to advertisements posted on message boards, social media, email and a university research participant pool (SONA). They were aged 18 years or above with a BMI >16.5 [consistent with a lower average observed in similar studies of this student population; see [23], and no reported history of eating disorder, neurological disease, or brain damage. Exclusion criteria were a history of psychiatric illness, substance abuse or dependency, or a first-degree relative with an eating disorder]. We excluded from analysis (see ‘Data Analysis’) participants who failed to complete key measures (i.e. the BART or cosmetic surgery measure; *n* = 27). This resulted in 265 participants who reported no previous cosmetic procedures and 10 who reported having undergone such procedures. A further 70 participants who reported no previous cosmetic procedures and 14 who reported having undergone such procedures sample were recruited at a public science event (*n* = 84). Our analyses were based on whether or not participants had undergone a cosmetic procedure, as described below (see ‘Data Analysis’). Demographic information based on these analyses is reported in Table 1.

The study was approved by an Institutional Research Ethics Committee (Project Number: 11781/001), and all participants gave written, informed consent prior to taking part.

### Main Measures

#### Demographic Information and Cosmetic History

We recorded participant age, ethnicity, height, and weight (for calculation of body mass index; BMI), and whether they had previously undergone any cosmetic surgery or procedures (with response options: ‘yes’, ‘no’, and ‘prefer not to say’).

#### Acceptance of Cosmetic Surgery Scale [ACSS; 26]

The ACSS is a validated [26] assessment of both general cosmetic surgery attitudes and the willingness to undergo cosmetic surgical procedures. Fifteen items are rated on a 7-point Likert scale, ranging from 1 (‘Strongly Disagree’) to 7 (‘Strongly Agree’). Three dimensions are assessed: Interpersonal (i.e. self-oriented beliefs about the benefits of cosmetic surgery), Social (i.e. social motivations for having cosmetic surgery), and Consider (e.g. how likely an individual would be to undergo cosmetic surgery). A global score is computed (and was used as predictor variable in the current study) from the combined average of the three subscale scores. Higher scores indicate a greater acceptance of cosmetic surgery. Total score Cronbach’s alpha in this study = 0.84.

#### Eating Disorder Examination Questionnaire [EDE-Q; 27]

The EDE-Q is a self-report measure of eating disorder symptoms, derived from the clinician-administered Eating Disorder Examination [EDE; 28]. Its psychometric properties have been extensively studied (see e.g. [29]), and it is one of the most extensively used assessments of disordered eating. Items assess attitudes, feelings, and behaviours related to eating and body image experienced over the past 28 days. Each item is scored on a 7-point scale representing the frequency of specific behaviours, with higher scores reflect greater eating-related pathology. The EDE-Q includes four subscales (i.e. Restraint, Eating Concern, Shape Concern, and Weight Concern), and a Global score which an average based on the four subscales. In the current study we analysed the impact of both Global symptoms and eating restraint specifically, given our previous findings relating this particular aspect of eating disorder symptomology to risk taking [23]. Global score Cronbach’s alpha in this study = 0.91

#### Barratt Impulsivity Scale [BIS-11; 30]

The BIS-11 contains 30 items assessing three aspects of impulsiveness: (1) Motor Impulsiveness—i.e. acting without thinking, (2) Non-Planning Impulsiveness—i.e. a lack of forethought, and (3) Attentional Impulsiveness—i.e. an inability to focus attention or concentrate. Each item is rated on a 4-point scale with 4 indicating the most impulsive response (‘Rarely/Never’ = 1; ‘Almost Always/Always’ = 4). Scores for the three sub-scales, as well as a total score (used as predictor variable in the current study), are obtained by summing the relevant item responses. The higher the summed score, the higher the level of impulsiveness (minimum global score = 30, maximum = 120). Psychometric data for the BIS-11 indicate good convergent validity, test−retest reliability, and internal consistency [31]. Total score Cronbach’s alpha in this study = 0.84.

#### Balloon Analogue Risk Task [BART; 15]

**Fig. 1:**
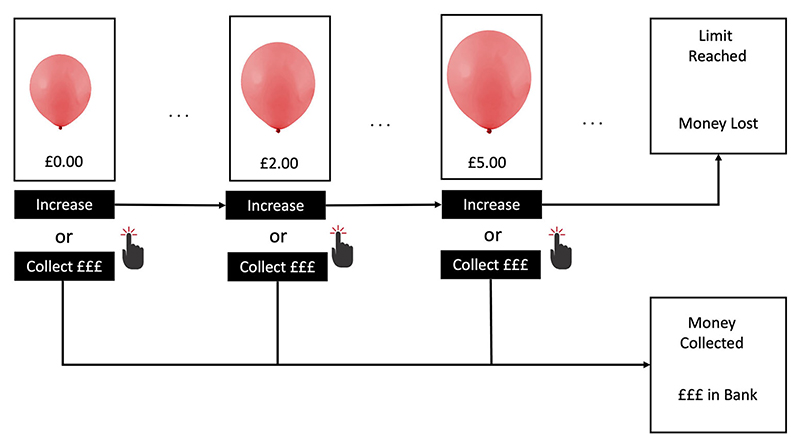
The Balloon Analogue Risk Task. *Note:* Each trial consists of a series of up to 116 stimuli which increase in size (‘inflate’) when a button is clicked (‘pumped’). For each balloon stimulus the participant makes a decision to either click the ‘Inflate’ button to win money (£0.05) and increase the size of the balloon, or collect any money earned so far into a permanent Bank and proceed to the next trial. The balloon stimulus reaches a limit at a point unknown to the participant (i.e. the loss limit/burst point), at which point the any money not collected during the trial is lost and the next trial begins.

The BART (see Fig. 1) is one of the most widely used measures of real-world risk taking [15]. The task involves participants ‘pumping’ a virtual balloon (by clicking a button) in order to win money. Each pump increases the size of the balloon and allows participants to win a small amount of money (i.e. £0.05), but at some point unknown to the participant, the balloon will burst. If participants ‘cash out’ the money they have earned into a permanent bank before the balloon bursts, they win the virtual money; however, if the balloon bursts beforehand they lose the money from that trial. The number of trials (balloons) and probability of burst per trial can be controlled by the experimenter. In the current study, participants completed 20 trials (balloons). The probability of burst for each trial was decided using an array of 116 numbers [15], with the number 1 designated as the balloon bursting. On each pump, a number was randomly selected from the array without replacement. Thus, with each click the probability of burst increases (i.e. 1/116 on first pump, 1/115 on second pump, etc.). Participants received standardised written instructions prior to completing the BART. At no point was the maximum number of pumps possible or probability of a burst mentioned. The number of trials (balloons) remaining, amount of money accrued on the current trial, and total amount of money in the permanent bank remained on screen, thereby allowing us to examine learning rates based on feedback.

#### BART Behavioural Measures

Although several behavioural measures can be derived from the BART [32], in the present study we calculated a widely used and recommended measure of explicit behavioural risk taking (our primary outcome variable): the number of pumps on trials in which the participant collected the accumulated money before the balloon burst (i.e. *number of pumps*).

### Supplementary Control Measures

We assessed body image disturbances [BIDQ; 33], depression anxiety and stress [DASS-21; 34], and obsessive-compulsive symptoms [short version, OCI-R; 35] in a sub-sample of participants, and used these as controls in our analyses (see ‘Data Analysis’ section). Full details of these measures are provided in supplementary materials.

### Procedure

Participants received an information sheet and completed a consent form prior to taking part, after which they completed the BART and questionnaires using a laptop computer. Participants who were tested in the laboratory at UCL completed all questionnaire measures, but those who took part at the public event completed a subset of these questionnaires (including only the BIS-11 and EDE-Q) due to time limitations. Completion of the study took approximately 30 min. Upon completion participants were thanked, debriefed, and given any compensation owed (i.e. either a small monetary reward for participants completing the study at UCL, or a small science-themed gift [brain-shaped eraser] at the public science event where monetary rewards were not permitted).

### Computational Modelling

We used computational modelling to understand the latent processes driving observable behaviour on the BART. Firstly, we examined the role of uncertainty (i.e. decisions made when both the outcome and the probability distribution are *unknown*) and risk (i.e. when participants have gained some knowledge of the outcome probabilities) [see 21, 22 for an in-depth discussion]. We developed this model by adapting an existing model of the BART [36]. The model assumes that in earlier trials of the BART decision making is driven by higher *uncertainty* and exploration to reduce uncertainty (exploration stage), while in the later trials uncertainty has reduced and *risk taking* drives behaviour (exploitation stage). Participants are assumed to hold a belief in the probability that they will reach the maximum limit (loss limit) during each of these two stages, and that a transition from exploration to exploitation occurs at a specific moment (threshold). Thus, the model has three parameters: (a) *Prior Probability of Loss Belief* (loss belief during uncertainty/exploration), (b) *Posterior Probability of Loss Belief* (loss belief during risk/exploitation), and (c) *Threshold* (the trial at which the transition from Exploration to Exploitation takes place).

Secondly, we examined whether (pumping) behaviour was driven by *risk* aversion or *loss* aversion, using the Exponential-Weight Model (EW model) [37, 38]. The EW model describes how sequential decisions are made during the BART, assuming that participants have a belief about the probability that the balloon will reach the maximum (loss limit), and that this belief is updated during the task through learning and evaluation that involves five parameters. Two of the model’s parameters are of relevance to the current study: *risk aversion* (*ρ*) indicates an individual’s sensitivity to the value of reward change, such that individuals with higher risk avoidance take less risk to get the same amount of reward. *Loss aversion* (*λ*) indicates an individual’s sensitivity to negative outcomes, such that potential loss is perceived as more severe at higher *λ*.

For both sets of modelling, we tested the fit (measuring the discrepancy between observed data and model predictions, while penalising for model complexity) and compared the models using Maximum Likelihood Estimation (MLE), BIC, and AIC. We selected the winning model by comparing these models with baseline models that assume no change in uncertainty, nor any influence of loss avoidance (respectively), in the participants’ behaviour. We then validated the recovered parameters by looking into their distributions and by using them in our model to generate simulations of behaviour on the BART which were compared to the actual participant BART behaviour [see Supplementary Materials for full details and results of this process, and 13 for descriptions of these different steps]. We subsequently used the parameters of the winning models in our analyses (see Analysis below, and Table 2 for a summary of computational parameters).

### Data Analysis

We first ran descriptive statistics (see Table 1) and zero-order correlations (see Supplementary Materials) with our main independent variables to examine for possible collinearity. To orthogonalize independent variables with suspected collinearity (e.g. ACSS, EDE-Q Global, and BIS-11 scores) we used the *fa* function from the *psych* R package which performs the *principal factor* solution. The orthogonalization gave results where each one of the initial variables had a reliable 1-to-1 correspondence to one resulting factor variable (loadings were higher than 0.98, average analysis complexity of 1, equal proportion of explained variance, and Tucker Lewis Index of factoring reliability of 1). This indicates that the new factors captured the variance explained by the initial variables while being orthogonal to each other, thus addressing the issues of collinearity that were present in the initial variables. We used these orthogonalized factor scores rather than the raw scores in all analyses.

#### Risk Taking in Women without Existing Cosmetic Procedures

Our first set of analyses used the data collected from women tested in the laboratory at UCL (*n* = 265) who had not undergone any cosmetic surgery (see Table 1). A series of linear mixed-effect models (LMM) examined if cosmetic surgery attitudes and behaviours (ACSS total score) predict risk taking (number of pumps on the BART). In our main analyses, we included age, BMI, eating disorder symptoms (Global EDE-Q score), and impulsiveness (BIS-11) as fixed effects (covariates), and Experimenter as a random effect to account for possible variability due to multiple experimenters. We ran this same analysis substituting Global EDE-Q with EDE-Q Restraint score, given previous evidence of its specific influence on risk taking [23]. We also carried out a series of control analyses in this sample where measures of body image concerns (BID-Q), obsessive-compulsive symptoms (OCI-R), and mood (DASS-21) were available, examining the influence of each control variable on the relationship between acceptance of cosmetic surgery (ACSS) and risk taking.

We then used computational modelling to examine whether pumping behaviour varies when making decisions under uncertainty versus risk and to disentangle the underlying role of risk versus loss aversion in participants’ decision making. We entered the same set of predictor variables (i.e. age, BMI, ACSS total, BIS-11, and EDE-Q Global/Restraint) in a series of LMMs, using as dependent variables the three parameters of our computational model of uncertainty versus risk: (1) prior probability of burst belief (i.e. burst belief during the initial exploration phase of the task), (2) posterior probability of burst belief (i.e. burst belief during the later exploitation phase of the task), and (3) threshold (i.e. the trial at which the transition from exploration to exploitation takes place). We then examined whether (pumping) behaviour was driven by *risk* aversion or *loss* aversion. We entered these same variables again as predictors in two LMMs, using as dependent variables the parameters from our computational modelling of risk versus loss aversion: (1) risk aversion (*ρ*) and (2) loss aversion (*λ*). Finally, we examined whether observed effects were modified by our control variables.

#### Risk Taking in Women with Existing Cosmetic Procedures

We conducted a separate analysis comparing all women who reported having undergone cosmetic surgery (including those tested at the laboratory and the public event; ‘Cosmetic Yes’ group; *n* = 24) to women recruited at the public event who had never undergone cosmetic surgery (‘Cosmetic No’ group), and classified as either ‘high’ (*n* = 18) or ‘low’ (*n* = 17) acceptance of cosmetic surgery using their ACSS total scores (i.e. high ACSS = score above the 75th percentile; low ACSS = score below the 25th percentile), in order to have more balanced comparison groups (see Table 1). We classified participants as having undergone cosmetic surgery based on an affirmative answer to questions regarding their past engagement with cosmetic procedures (see Measures). Subsequent analyses of behavioural data and computational modelling were performed using Group (Cosmetic Yes vs. High ACSS vs. Low ACSS), BMI and age (but no other covariates or control analyses as these were not collected from the public event participants due to time constraints) as predictor variables. Where samples drawn from different recruitment locations (UCL vs. public event) were combined for analysis, we included ‘Study’ (i.e. experimenter) as a Random variable in our analysis to account for study effects.

#### Analytical Procedures

To fit our linear mixed-effects models (LMM) to data we used the *lmer* R function of the *lme4* package. To generate the results table for our lmer-fitted models we used the *tab_model* function of R from the *sjPlot* package. In this package, for linear mixed models (lmerMod-objects), the computation of p-values (if p.kr = TRUE) is based on conditional *F*-tests with Kenward−Roger approximation for the df, using the *pbkrtest* package. In results we present for each variable: (i) the slope *b* and it 95% CI, (ii) *p*-value, and (iii) the *R*^2^-marginal increase, a measure of effect size that allows the unique contribution of each predictor to be identified the (i.e. variance explained with variable in the full model—variance explained without predictor in the model). Where the total variance explained by random effects (ICC) was <.001 we removed the variable from the model to avoid having redundant random effects.

## Results

### Risk Taking in Women Without Existing Cosmetic Procedures

#### Descriptive Statistics

Descriptive statistics (*M* and SD) are reported in Table 1. Correlations between variables and reliability statistics are reported in Supplementary Materials.

#### BART Performance

**Fig. 2:**
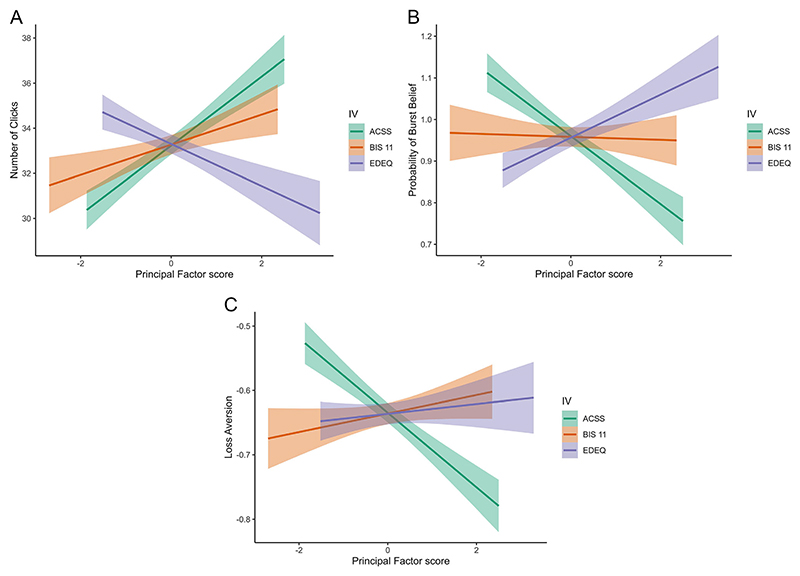
Results in women with no history of cosmetic surgery. *Note:* **A** regression slopes for predictors of behavioural risk taking (number of pumps). **B** Regression slopes for predictors of decision making under risk phase of the BART. **C** Regression slopes for predictors of loss aversion.

Results of the LMM that included age, BMI, acceptance of cosmetic surgery (ACSS), eating disorder symptoms (Global EDE-Q), and impulsivity (BIS-11) as predictors of risk-taking (pumping) behaviour indicated that acceptance of cosmetic surgery, impulsivity, and age were all significant predictors of the total number of pumps (see Figure 2, Panel A). Of particular interest to our current hypothesis, as acceptance of cosmetic surgery increases, the total number of balloon pumps participants make also increases (*b* = 1.68, *p* =.033, 95% CI [0.14−3.22]; *R*^2^_Marginal Increment_ = 0.013). Likewise, as impulsivity increases so does the total number of balloon pumps (*b* = 1.91, *p* =.037, 95% CI [0.11−3.71]; *R*^2^_Marginal Increment_ = 0.012). By contrast, increasing age predicts fewer balloon pumps (*b* = − 0.34, *p* =.006, 95% CI [− 0.59−− 0.10]; *R*^2^_Marginal Increment_ = 0.011). Eating disorder symptoms, while not statistically significant, showed an effect direction consistent with our previous research looking at eating restraint [23], such that increasing eating disorder symptoms predicts a decrease in pumping (*b* = − 1.21, *p* =.112, 95% CI [− 2.71−0.28]; *R*^2^_Marginal Increment_ = 0.004). Running these same analyses with eating restraint as a covariate instead of global eating disorder symptoms produced the same pattern of results, and including our control variables did not substantially reduce the observed effect of cosmetic surgery attitudes on risk-taking (pumping) behaviour (see Supplementary Materials).

#### Uncertainty Versus Risk

Our LMM that included age, BMI, acceptance of cosmetic surgery (ACSS), eating disorder symptoms (Global EDE-Q), and impulsivity (BIS-11) as predictors of a participant’s belief about the probability that a burst will occur (herein: burst belief) during the initial, uncertainty (exploration) phase of the task, identified only age as a significant predictor (*b* = 0.02, *p* =.015, 95% CI [0.00−0.03]; *R*^2^_Marginal Increment_ = 0.014). Running this analysis using eating restraint as a covariate instead of global eating disorder symptoms produced the same pattern of results.

A LMM using the same main predictor and outcome variables in relation to the later, risk-taking (exploitation) phase of the task indicated that (in addition to age; *b* = 0.03, *p* <.001, 95% CI [0.01−0.04]; *R*^2^_Marginal Increment_ = 0.045), acceptance of cosmetic surgery (*b* = − 0.08, *p* =.040, 95% CI [− 0.17−0.00]; *R*^2^_Marginal Increment_ = 0.011), and eating disorder symptoms (*b* = 0.08, *p* =.044, 95% CI [0.00−0.16]; *R*^2^_Marginal Increment_ = 0.013) were significant predictors of burst belief (see Fig. 2, Panel B). As acceptance of cosmetic surgery increases, burst belief decreases. By contrast, as eating disorder symptoms increase, burst belief increases. Again, running these analyses using eating restraint as a covariate instead of global eating disorder symptoms produced the same overall pattern of results, but with acceptance of cosmetic surgery showing a trend (*b* = − 0.08, *p* =.055, 95% CI [− 0.16−0.00]; *R*^2^_Marginal Increment_ = 0.009), and eating restraint having a slightly larger and more significant effect (*b* = 0.10, *p* =.015, 95% CI [0.02−0.17]; *R*^2^_Marginal Increment_ = 0.019). Including our control variables did not substantially reduce the observed effect of cosmetic surgery attitudes on burst beliefs (see Supplementary Materials).

#### Avoidance of Loss Versus Risk

In two final analyses we used separate LMMs to examine whether the behaviour of participants was driven by avoidance of loss or risk. Both analyses included our standard set of predictor variables (i.e. age, BMI, acceptance of cosmetic surgery, eating disorder symptoms/eating restraint, and impulsivity), with either loss (*λ*) or risk (*ρ*) aversion used as outcome variables. Results of the LMMs predicting loss aversion (*λ*) revealed acceptance of cosmetic surgery to be the sole, significant predictor of *loss aversion* (see Fig. 2, Panel C; *b* = − 0.09, *p* =.037, 95% CI [− 0.17−− 0.01]; *R*^2^_Marginal Increment_ = 0.02 when including global eating disorder symptoms; and *b* = − 0.09, *p* =.040, 95% CI [− 0.17−0.00]; *R*^2^_Marginal Increment_ = 0.02 when including eating restraint). As acceptance of cosmetic surgery increases, loss aversion decreases. Including our control variables in these statistical models did not substantially reduce this relationship (see Supplementary Materials). By contrast, none of our main variables were significant predictors of *risk aversion* (*ρ*).

## Risk Taking in Women with Existing Cosmetic

### Procedures

#### BART Performance

**Fig. 3:**
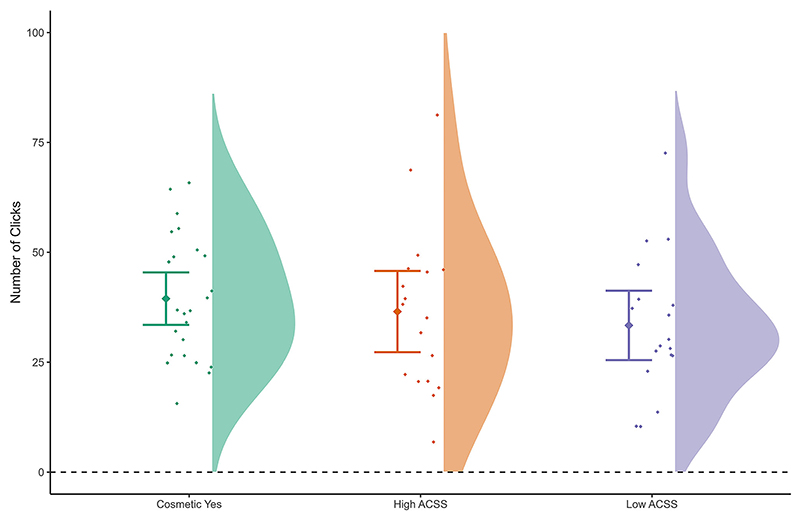
Behavioural risk taking (pumping) results in women with a history of cosmetic surgery.

The LMM that included age, BMI, and Group (Cosmetic Yes vs. High ACSS vs. Low ACSS), as predictors of risk-taking behaviour, indicated that BMI was a significant predictor of the total number of pumps, with higher BMI predicting greater risk taking (*b* = 0.79, *p* = .039, 95% CI [0.04−1.53], *R*^2^_Marginal Increment_ = 0.04). Group showed a statistical trend and an effect direction consistent with that found in our first set of analyses in women who had not undergone cosmetic surgery, i.e. women who had undergone cosmetic surgery took more risk than women who scored low in their acceptance of cosmetic surgery (Figure 3; *b* = 7.85, *p* = .099, 95% CI [− 1.46−17.16], *R*^2^-_Marginal Increment_ = 0.025), and including our control variables did not substantially reduce this effect (see Supplementary Materials).

#### Uncertainty Versus Risk

Using these same predictor variables to examine burst beliefs during uncertainty (aka phase 1: exploration) and risk (aka phase 2: exploitation) showed that BMI (*b* = − 0.06, *p* = .003, 95% CI [− 0.10−− 0.02], *R*^2^-_Marginal Increment_ = 0.148) and Group were significant predictors when decisions were made under conditions of risk, but not uncertainty. The direction of the Group effect was consistent with that observed in our analyses of women without a history of cosmetic surgery: women who had undergone cosmetic surgery and the subgroup of women who had high acceptance of cosmetic surgery held a significantly lower belief that the balloon would burst compared with women with a low acceptance of cosmetic surgery (cosmetic yes: *b* = − 0.61, *p* = .020, 95% CI [− 1.11−− 0.10], *R*^2^_Marginal Increment_ = 0.107; ACSS high: *b* = − 0.71, *p* = 0.015, 95% CI [− 1.27−− 0.14], *R*^2^-_Marginal Increment_ = 0.107). Including our control variables did not substantially reduce this effect (see Supplementary Materials).

#### Avoidance of Loss Versus Risk

Our examination of loss versus risk avoidance indicated that age (*b* = − 0.03, *p* = 0.028, 95% CI [− 0.05−0.00], *R*^2^_Marginal Increment_ = 0.088) and BMI (*b* = − 0.05, *p* = .003, 95% CI [− 0.09−− 0.02], *R*^2^_Marginal Increment_ = 0.157) were significant predictors of loss avoidance. Group did not significantly predict loss or risk avoidance; however, the observed effects (*b*-values) were consistent with those observed in our analyses of women without a history of cosmetic surgery, with increased acceptance of cosmetic surgery (i.e. cosmetic yes and high ACSS groups) being associated with less loss avoidance (cosmetic yes: *b* = − 0.04, *p* = 0.861, 95% CI [− 0.52−0.44], *R*^2^_Marginal Increment_ = 0.043; ACSS high: *b* = − 0.36, *p* = 0.142, 95% CI [− 0.85−0.13], *R*^2^_Marginal Increment_ = 043) but no effects in relation to risk avoidance (cosmetic yes: *b* = 0.00, *p* = 0.609, 95% CI [− 0.02−0.01], *R*^2^_Marginal Increment_ = 007; ACSS high: *b* = 0.00, *p* = 0.766, 95% CI [− 0.02−0.01], *R*^2^_Marginal Increment_ = 007). Including our control variables in these statistical models did not substantially reduce the observed effects (see Supplementary Materials).

## Discussion

We used experimental and computational methods to examine the relationship between cosmetic surgery attitudes and history, and factors that drive risk-taking behaviour. We predicted that greater acceptance of cosmetic surgery, or having undergone a cosmetic procedure, would predict greater risk-taking behaviour (balloon pumping). Our results support this hypothesis, finding significantly greater risk-taking behaviour in women with greater acceptance of cosmetic surgery, as well as (a statistical trend for) increased risk taking in a smaller sample of women with a history of cosmetic procedures. Furthermore, using a previously validated method of computationally modelling performance on the risk-taking task [23, 38], we were able to draw conclusions regarding the latent (hidden) parameters that may drive participants’ behaviour. More specifically, we were able first to determine that, as predicted, the observed increased risk taking related to how participants in both samples made decisions when the task involved ‘risk’ (knowing the probabilities of loss), and not ‘uncertainty’ (not knowing the probabilities of loss), or the point at which people switch from uncertainty to risk. Secondly, contrary to our predictions, we found a reduction in loss rather than risk aversion in women with increased acceptance of cosmetic surgery and women with a history of cosmetic procedures. In other words, our findings suggest that cosmetic surgery candidates may place less emphasis on what are the possible losses at stake (i.e. their choices are driven by reduced loss aversion) and, thus, make more risky choices. Moreover, we found that the observed relationships between cosmetic surgery attitudes and risk taking were over and above any effects of age, BMI, eating disorder symptomology and impulsivity, and not reduced by the inclusion of obsessive-compulsive symptoms, body image concerns, or mood variables in the analyses.

Understanding the motivating factors that drive cosmetic surgery acceptance is important, as cosmetic procedures carry inherent medical risks (e.g. infection) and have potentially negative psychological outcomes (e.g. disappointment) [2] and psychosocial consequences (e.g. negative perceptions from people) [3]. Our findings add to a growing body of empirical work that has attempted to understand the demographic, personal, and clinical factors that predict cosmetic surgery acceptance or the intention to undergo surgery [7, 8, 10, 39]. However, no study to date has used experimental and computational methods of decision making to characterise the underlying cognitive-motivational factors associated with cosmetic surgery attitudes and choices. These methods complement existing studies, by allowing participants’ performance when making decisions involving risk to be measured and the underlying drivers of observed behaviour to be analysed.

Our finding of greater risk-taking behaviour driven by reduced loss aversion in women with greater acceptance of cosmetic surgery is previously undocumented and important for clinicians working with individuals seeking cosmetic procedures. Individuals who are considering cosmetic surgery may be more inclined to make risky decisions, even when presented with information about the likelihood of adverse outcomes. In particular, our results suggest that cosmetic surgery candidates may be less influenced by (i.e. give less psychological weight and attention to) information about the possible adverse outcomes (losses) associated with surgery. Hence, policies regarding the presentation of the risks of cosmetic surgery may need to place particular emphasis not only on how risky, or uncertain the various procedures are, but what are the particular negative consequences, or side effects that an individual may face. Based on these characteristics, people considering cosmetic surgery need to understand the risks and potential losses better, and research that investigates how best to provide such information so that people can imagine it as relevant to their ‘future self’ is needed. This might include examining different ways of verbalising or visualising risk- and loss-related information, and assessing whether giving information using different methods helps those with high risk-taking tendencies to consider the potential losses associated with cosmetic surgery to a greater extent.

Further research also needs to examine the extent to which our findings generalise to different populations. Future research needs to examine risk taking associated with cosmetic surgery attitudes and intentions in men, since different standards of physical attractiveness apply to men and women [40], and existing research suggests gender differences in risk taking [41]. Socioeconomic, demographic, cultural, and ethnic factors also influence attitudes and drives towards cosmetic surgery [4, 7, 24, 42], and research is needed to examine whether risk-taking tendencies interact with such factors to influence cosmetic surgery attitudes and intentions. Our sample of women with a history of cosmetic procedures was also relatively small, and although the effects found in this sub-sample were consistent with the (statistically) significant effects found in the larger sample of women whose attitudes towards cosmetic surgery were measured, future research is needed to confirm our findings in individuals with a history of cosmetic surgery.

Finally, we note some strengths and limitations of using the BART. The BART is a widely used and validated measure of risk taking, and a good proxy of real-world risk-taking behaviour [15, 16, 43]. Moreover, our use of computational modelling allowed us to overcome known limitations of the BART [22] and identify the latent variables that drive observable behaviour. However, an interesting area for future research is to examine how more salient, body-based rewards might influence risk taking. In recent research involving women with restrictive eating (including women with anorexia nervosa), we found that body-based stimuli with high social−motivational salience (i.e. images of women of varying degrees of ‘thinness’) moderated risk-taking behaviour, with greater risk taking observed in women with greater restrictive eating when decisions were linked to an thinner versus a larger body [23]. An interesting area for future research would be to examine whether cosmetic surgery-related risk taking is even greater when the potential reward is obtaining their ideal body after surgery, such as when specific, desired body modifications (e.g. nose, lip, or eye reshaping) are at stake.

In summary, our results suggest that when making decisions, women seeking cosmetic procedures may be less sensitive to possible losses and may thus take more risky decisions, and that a greater emphasis should, therefore, be placed on communicating potential losses (what one stands to lose) rather than just the associated risks (how likely one is to suffer some loss) of cosmetic procedures.

## Supplementary Material

Supplementary material

## Figures and Tables

**Table 1:** Demographic information and descriptive statistics for key behavioural and control variables. Samples are grouped and summarised according to their corresponding analysis of (1) women with no history of cosmetic surgery and (2) women with a history of cosmetic surgery vs. women with low ACSS vs. high ACSS scores.

	Sample 1	Sample 2		
	No history of cosmetic surgery	History of cosmetic surgery	High ACSS	Low ACSS
N	265	24	18	17
Age	23.7 (6.1)	29.7 (8.9)	25.4 (5.5)	32.3 (10.6)
BMI	22.2 (4.2)	23 (5.9)	21.3 (3.1)	22.4 (4.1)
*Ethnicity*				
Asian	52%	19%		
Black	4%	0%		
Mixed	3%	0%		
White	32%	69%		
Other	9%	12%		
ACSS	3.1 (1.2)	4.7 (1)	4.2 (0.5)	1.7 (0.4)
EDE-Q (Global Score)	1.7 (1.3)	1.9 (1.2)	1.8 (1.1)	1.1 (1)
BIS-11	68.1 (11.5)	–	–	–
Total number of pumps	31.5 (15.6)	37.5 (19)	32.7 (20.7)	28.8 (17)

− (dash) indicates measure not collected.

**Table 2:** Glossary of key variables and computational parameters

Variable/computational parameter	Definition
Risk-taking behaviour/behavioural risk taking	Number of pumps on trials in which the participant collected the accumulated money before the balloon burst
Uncertainty/exploration (phase)	When both the outcome and the probability distribution are unknown
Risk/exploitation (phase)	When the outcome is unknown, but the outcome probabilities are known
Risk aversion *(ρ)*	An individual’s sensitivity to the value of reward change, such that individuals with higher risk avoidance take less risk to get the same amount of reward
Loss aversion (*λ*)	An individual’s sensitivity to negative outcomes, such that potential loss is perceived as more severe at higher *λ*

## Data Availability

Data and code used in the research are available via GitHub: https://github.com/katlaboratory/risktaking_cosmetic.

## References

[1] International Society of Aesthetic Plastic Surgery (2021). ISAPS-Global-Survey_2020pdf.

[2] Arnocky S, Piché T (2014). Cosmetic surgery as intrasexual competition: the mediating role of social comparison. Psychology.

[3] Bonell S, Murphy SC, Griffiths S (2021). Under the knife: unfavorable perceptions of women who seek plastic surgery. PLoS ONE.

[4] Alotaibi AS (2021). Demographic and cultural differences in the acceptance and pursuit of cosmetic surgery: a systematic literature review. Plast Reconstr Surg Glob Open.

[5] Haas CF, Champion A, Secor D (2008). Motivating factors for seeking cosmetic surgery: a synthesis of the literature. Plast Surg Nurs Off J Am Soc Plast Reconstr Surg Nurses.

[6] Milothridis P, Pavlidis L, Haidich A-B, Panagopoulou E (2016). A systematic review of the factors predicting the interest in cosmetic plastic surgery. Indian J Plast Surg.

[7] Brown A, Furnham A, Glanville L, Swami V (2007). Factors that affect the likelihood of undergoing cosmetic surgery. Aesthet Surg J.

[8] Furnham A, Levitas J (2012). Factors that motivate people to undergo cosmetic surgery. Can J Plast Surg J Can Chir Plast.

[9] Moser SE, Aiken LS (2011). Cognitive and emotional factors associated with elective breast augmentation among young women. Psychol Health.

[10] Park JS, Cho C-H (2010). Factors explaining college students’ intention to receive cosmetic surgery in the future: a structural equation modeling approach. J Med Mark.

[11] Swami V, Chamorro-Premuzic T, Bridges S, Furnham A (2009). Acceptance of cosmetic surgery: personality and individual difference predictors. Body Image.

[12] Ostaszewski P, Bąbel P, Swebodziński B (2013). Physical and cognitive effort discounting of hypothetical monetary rewards: physical and cognitive effort discounting. Jpn Psychol Res.

[13] Wilson RC, Collins AG (2019). Ten simple rules for the computational modeling of behavioral data. Elife.

[14] Odum AL (2011). Delay discounting: I’M A K, YOU’RE A K. J Exp Anal Behav.

[15] Lejuez CW, Read JP, Kahler CW (2002). Evaluation of a behavioral measure of risk taking: the balloon analogue risk task (BART. J Exp Psychol Appl.

[16] Lejuez CW, Aklin WM, Jones HA (2003). The balloon analogue risk task (BART) differentiates smokers and non-smokers. Exp Clin Psychopharmacol.

[17] Canning JR, Schallert MR, Larimer ME (2022). A systematic review of the balloon analogue risk task (BART) in alcohol research. Alcohol Alcohol.

[18] Mishra S, Lalumière ML, Williams RJ (2017). Gambling, risk-taking, and antisocial behavior: a replication study supporting the generality of deviance. J Gambl Stud.

[19] Bornovalova MA, Gwadz MA, Kahler C (2008). Sensation seeking and risk-taking propensity as mediators in the relationship between childhood abuse and HIV-related risk behavior. Child Abuse Negl.

[20] Lauriola M, Panno A, Levin IP, Lejuez CW (2014). Individual differences in risky decision making: a meta-analysis of sensation seeking and impulsivity with the balloon analogue risk task. J Behav Decis Mak.

[21] De Groot K, Thurik R (2018). Disentangling risk and uncertainty: when risk-taking measures are not about risk. Front Psychol.

[22] De Groot K (2020). Burst beliefs—methodological problems in the balloon analogue risk task and implications for its use. J Trial Error.

[23] Jenkinson P, Koukoutsakis A, Panagiotopoulou E (2023). Body appearance values modulate risk aversion in eating restriction. J Exp Psychol Gen.

[24] Frederick DA, Lever J, Peplau LA (2007). Interest in cosmetic surgery and body image: views of men and women across the lifespan. Plast Reconstr Surg.

[25] Henderson-King D, Brooks KD (2009). Materialism, sociocultural appearance messages, and paternal attitudes predict college women’s attitudes about cosmetic surgery. Psychol Women Q.

[26] Henderson-King D, Henderson-King E (2005). Acceptance of cosmetic surgery: scale development and validation. Body Image.

[27] Fairburn CG, Beglin SJ (1994). Assessment of eating disorders: interview or self-report questionnaire?. Int J Eat Disord.

[28] Cooper Z, Fairburn C (1987). The eating disorder examination: a semi-structured interview for the assessment of the specific psychopathology of eating disorders. Int J Eat Disord.

[29] Berg KC, Peterson CB, Frazier P, Crow SJ (2012). Psychometric evaluation of the eating disorder examination and eating disorder examination-questionnaire: a systematic review of the literature. Int J Eat Disord.

[30] Patton JH, Stanford MS, Barratt ES (1995). Factor structure of the Barratt impulsiveness scale. J Clin Psychol.

[31] Stanford MS, Mathias CW, Dougherty DM (2009). Fifty years of the barratt impulsiveness scale: an update and review. Pers Individ Differ.

[32] Schmitz F, Manske K, Preckel F, Wilhelm O (2016). The multiple faces of risk-taking: scoring alternatives for the balloon-analogue risk task. Eur J Psychol Assess.

[33] Cash TF, Phillips KA, Santos MT, Hrabosky JI (2004). Measuring “negative body image”: validation of the body image disturbance questionnaire in a nonclinical population. Body Image.

[34] Lovibond SH, Lovibond PF (1995). Manual for the depression anxiety stress scales.

[35] Foa EB, Huppert JD, Leiberg S (2002). The obsessive-compulsive inventory: development and validation of a short version. Psychol Assess.

[36] Wallsten TS, Pleskac TJ, Lejuez CW (2005). Modeling behavior in a clinically diagnostic sequential risk-taking task. Psychol Rev.

[37] Park H, Yang J, Vassileva J, Ahn W-Y (2019). Development of a novel computational model for the balloon analogue risk task: the exponential-weight mean-variance model. J Math Psychol.

[38] Park H, Yang J, Vassileva J, Ahn W-Y (2021). Development of a novel computational model for the balloon analogue risk task: the exponential-weight mean–variance model. J Math Psychol.

[39] Park LE, Calogero RM, Harwin MJ, DiRaddo AM (2009). Predicting interest in cosmetic surgery: Interactive effects of appearance-based rejection sensitivity and negative appearance comments. Body Image.

[40] Grogan S (2021). Body image: understanding body dissatisfaction in men, women, and children.

[41] Byrnes JP, Miller DC, Schafer WD (1999). Gender differences in risk taking: a meta-analysis. Psychol Bull.

[42] Sinno S, Lam G, Brownstone ND, Steinbrech DS (2016). An assessment of gender differences in plastic surgery patient education and information in the United States: are we neglecting our male patients?. Aesthet Surg J.

[43] Lejuez CW, Aklin WM, Zvolensky MJ, Pedulla CM (2003). Evaluation of the balloon analogue risk task (BART) as a predictor of adolescent real-world risk-taking behaviours. J Adolesc.

