# Supplementary material for: Greater Risk Taking in Cosmetic Surgery Acceptance and History: An Experimental and Computational Study
Source: Aesthetic Plast Surg. 2024 Mar 21;48(13):2561–72. doi: 10.1007/s00266-024-03910-9 (PMC7616178; doi:10.1007/s00266-024-03910-9)

# Supplementary Materials

## Supplementary Control Measures

### Body Image Disturbance Questionnaire (BIDQ)

The BIDQ comprises quantifies body image impairment in terms of body dissatisfaction, distress and dysfunction. Low scores indicate a low level of concern, no distress, or functional limitation, whereas high scores indicating a high level of concern, emotional distress, or impairment of function. Previous research [1, 2] has established the BIDQ as a valid assessment in non-clinical samples with good internal consistency and test-retest reliability.

### Depression Anxiety Stress Scales – 21-Item Version (DASS-21)

The DASS-21 is a set of three self-report scales designed to measure depression, anxiety and stress. Scores for each scale are calculated by summing the relevant subscale items, with higher scores indicating a greater occurrence of the specific dimension. The DASS-21 has demonstrated good psychometric properties, with good concurrent validity and internal consistency [3].

### Obsessive-Compulsive Inventory Short Version (OCI-R)

The OCI-R is a self-report questionnaire designed to assess a variety of obsessions and compulsions, including obsessing, washing, hoarding, ordering, checking and neutralizing behaviours. The outcome measure is a sum of all items, with a possible range from 0-72, and higher scores indicating greater obsessive-compulsive behaviours. The OCI-R has good to excellent internal consistency, test-retest reliability, and convergent validity [4].

**References**

1. Cash TF, Phillips KA, Santos MT, Hrabosky JI (2004) Measuring “negative body image”: validation of the Body Image Disturbance Questionnaire in a nonclinical population. Body Image 1:363–372. https://doi.org/10.1016/j.bodyim.2004.10.001

2. Cash TF, Grasso K (2005) The norms and stability of new measures of the multidimensional body image construct. Body Image 2:199–203. https://doi.org/10.1016/j.bodyim.2005.03.007

3. Antony MM, Bieling PJ, Cox BJ, et al (1998) Psychometric properties of the 42-item and 21-item versions of the Depression Anxiety Stress Scales in clinical groups and a community sample. Psychol Assess 10:176–181. https://doi.org/10.1037/1040-3590.10.2.176

4. Foa EB, Huppert JD, Leiberg S, et al (2002) The Obsessive-Compulsive Inventory: Development and validation of a short version. Psychol Assess 14:485–496. https://doi.org/10.1037/1040-3590.14.4.485

## Results using ACCSS Total – BIS11 Total and EDEQ Global as predictors


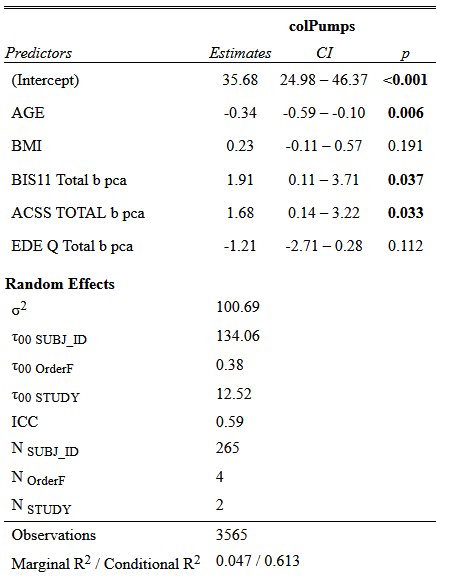

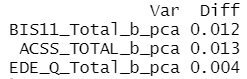


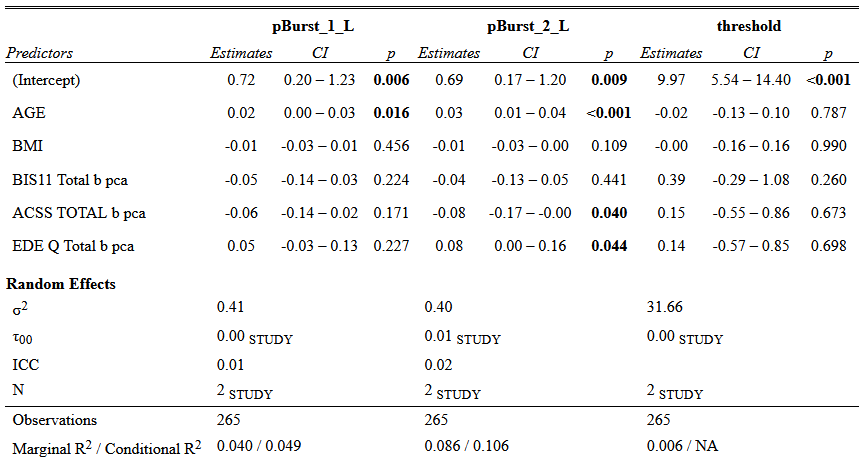


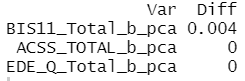

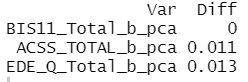

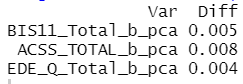


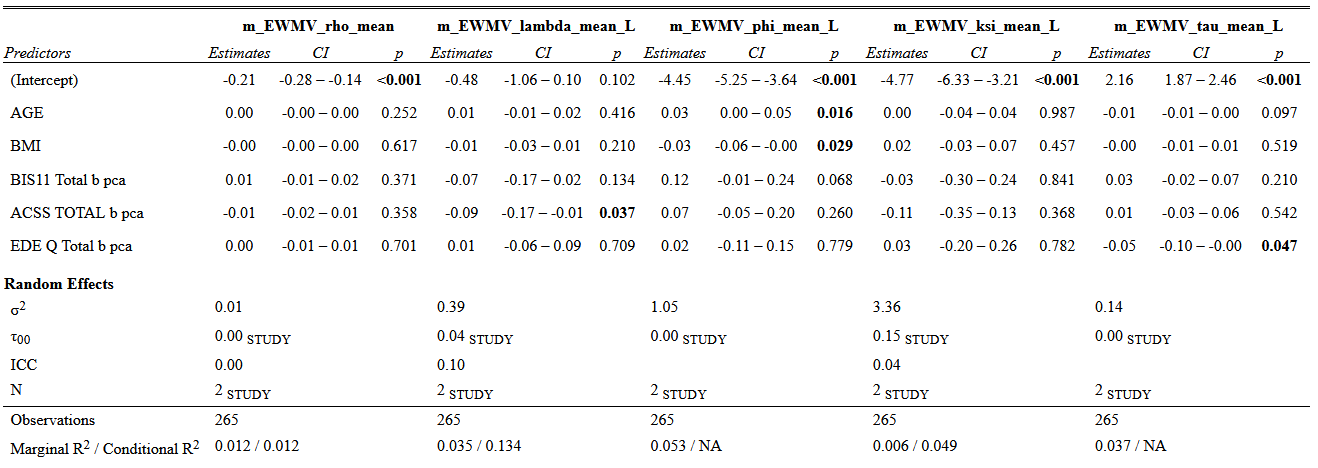

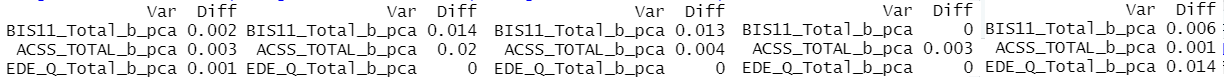


## Results using ACCSS Total – BIS11 Total and EDEQ Restraint as predictors


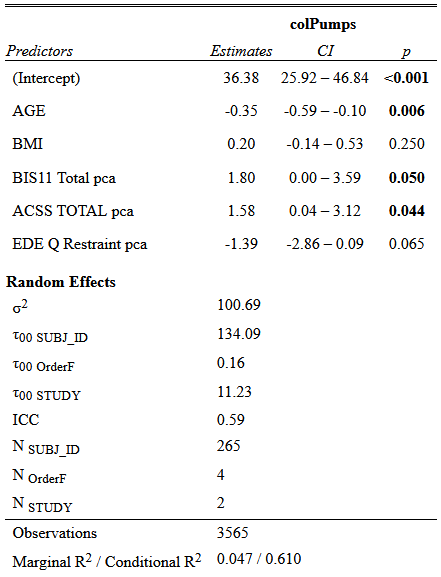

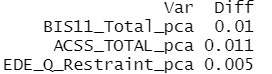


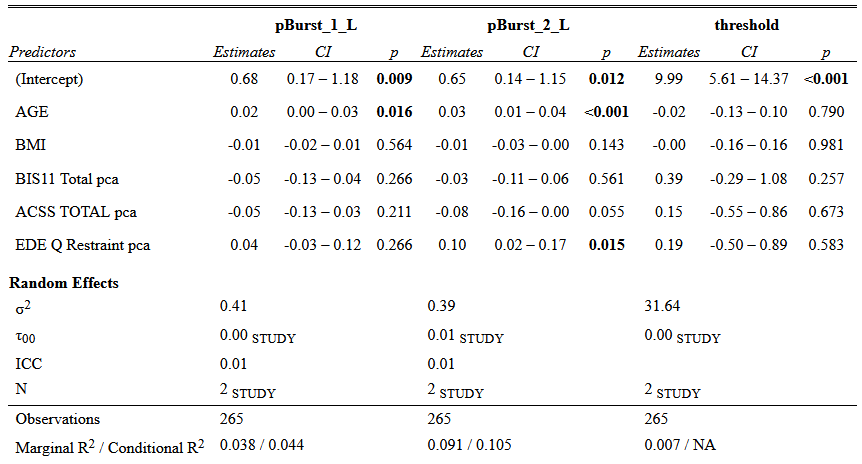


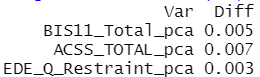

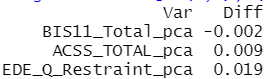

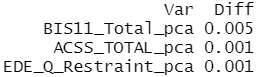


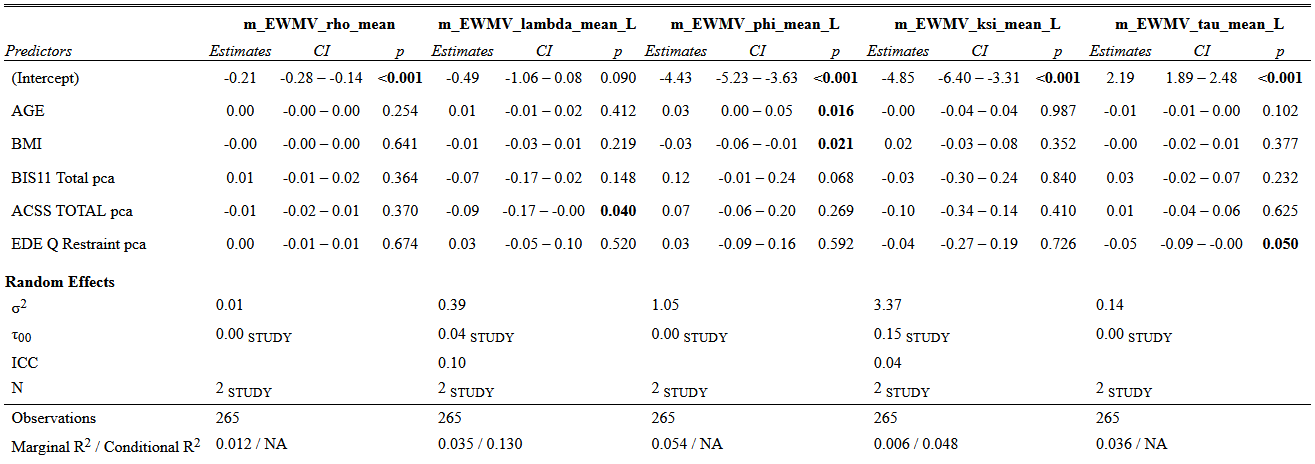


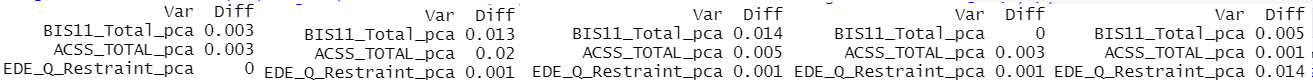


## Results for COSMETIC DONE


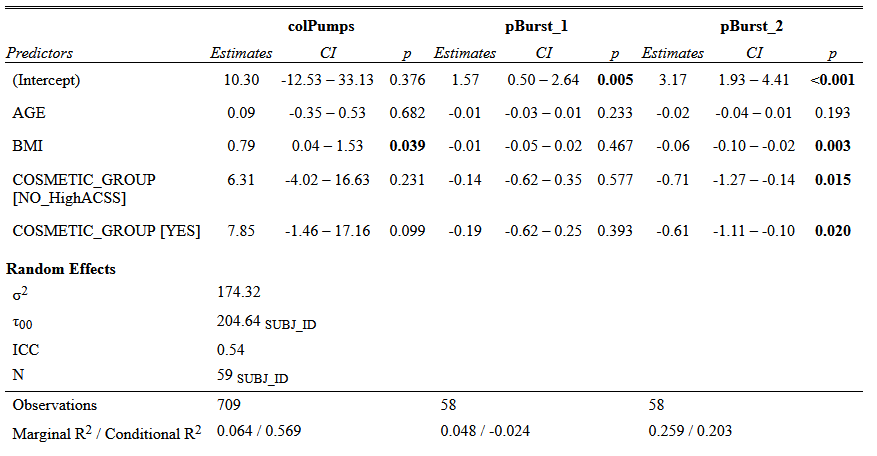


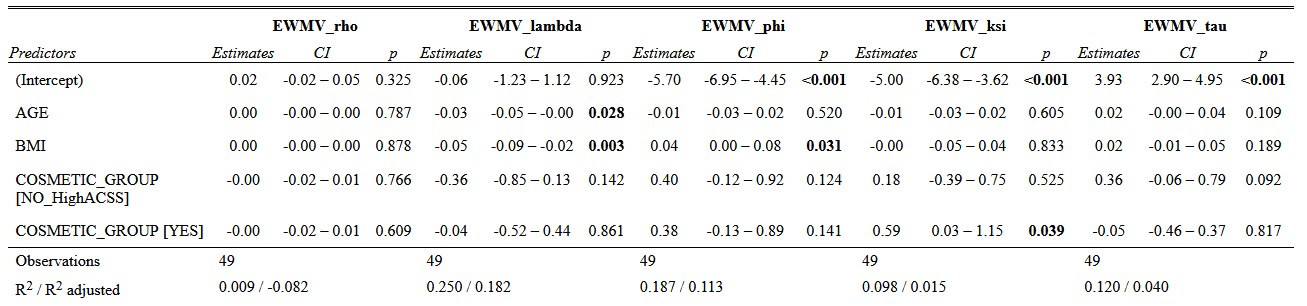


## Principal Factor Solution, Cronbach Alpha and Correlations for ACSS, EDEQ, and BIS11

### Cronbach Alphas

ACSS: 0.84

EDEQ: 0.91

BIS11: 0.84

### Correlations Matrix


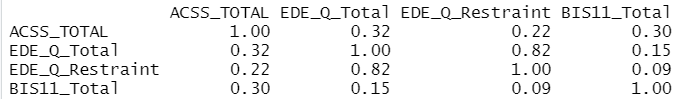


### Correlations P-Values


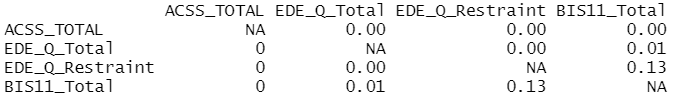


### Principal Factor Solution- ACSS, EDEQ Global, and BIS11


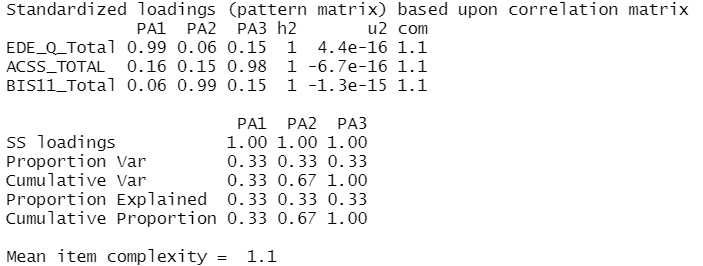


Tucker Lewis Index of factoring reliability = 1.05

### Principal Factor Solution- ACSS, EDEQ Restraint, and BIS11


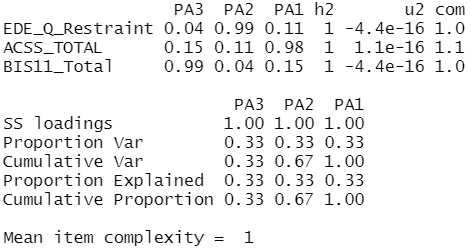


Tucker Lewis Index of factoring reliability = 1.072

## CONTROLS - Results using ACCSS Total – BIS11 Total and EDEQ Global as predictors plus Control Variables

Control variables DASS 21 (Anxiety, Depression and Stress), BIDQ Total, OCIR Total were sequentially used, one at a time, as covariates in addition to our 3 core predictors to investigate if our findings would be affected. We notice that in all cases the Effect Slopes remain very close to the slopes of the main findings. Small differences and lack of statistical significance were expected as the sample size of this analysis is almost half of that of the main analysis.

## CONTROLS - Results using ACCSS Total – BIS11 Total and EDEQ Restraint as predictors plus Control Variables

Same as the previous section.

## CONTROLS - Results using Cosmetic Done as predictor plus Control Variables

The main findings remain valid and statistically significant even after the inclusion of the covariates EDEQ Global and EDEQ Restraint and despite the small reduction in the sample size.


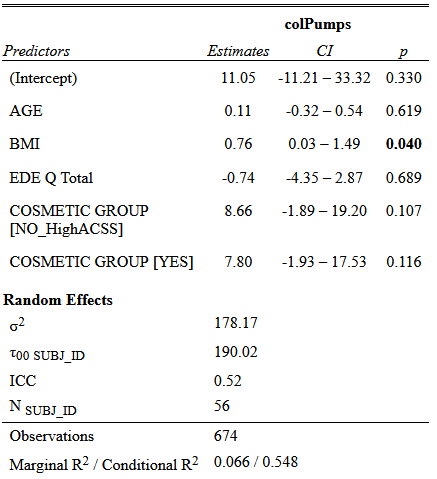


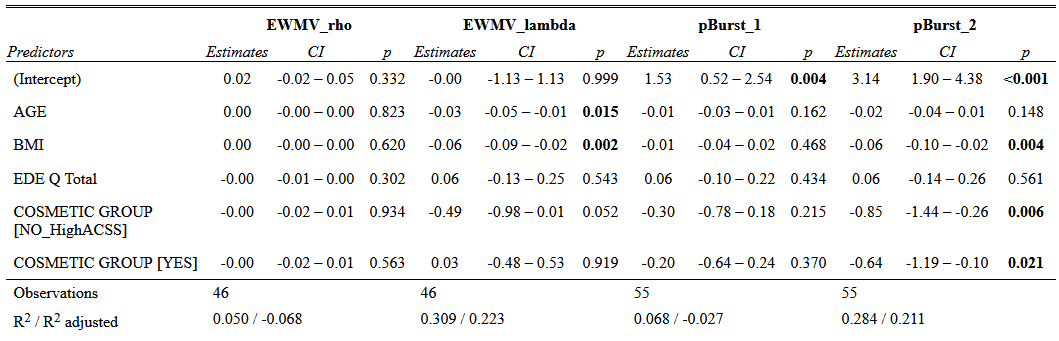


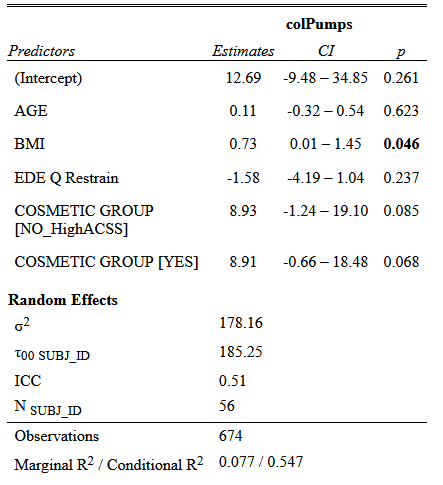

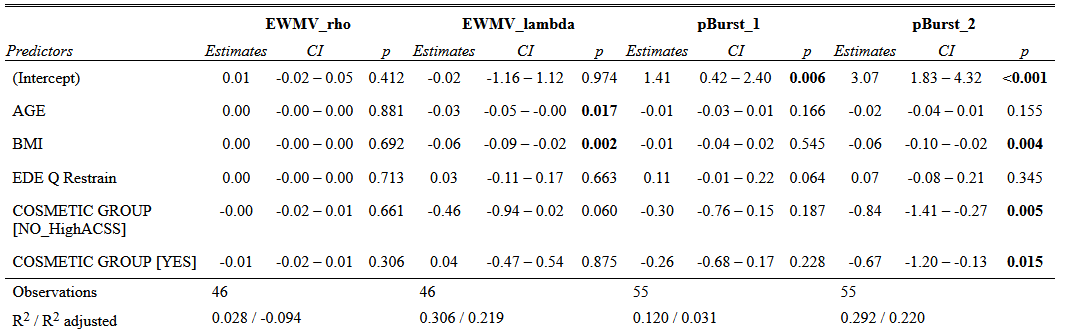


## Correlations

# Computational Modelling Overview

In this document, we perform model comparison between the Baseline model, the 2-Phase model and the EWMV model using Loglikelihood, AIC and BIC, and we investigate the parameter recovery of the 2-Phase and EWMV models, as well as the relationship between the 3 Probability of Burst Belief recovered parameters from the two models. Additionally, we used the recovered parameters of all subjects, to generate simulated trials for the two models and compare the results to the subject’s actual behavioural data.

# Model Comparison

The 2-Phase model is better than the Baseline model in all three criteria, whereas the EWMV model is better than the 2-Phase model in all criteria.

# EWMV Model Parameter Recovery


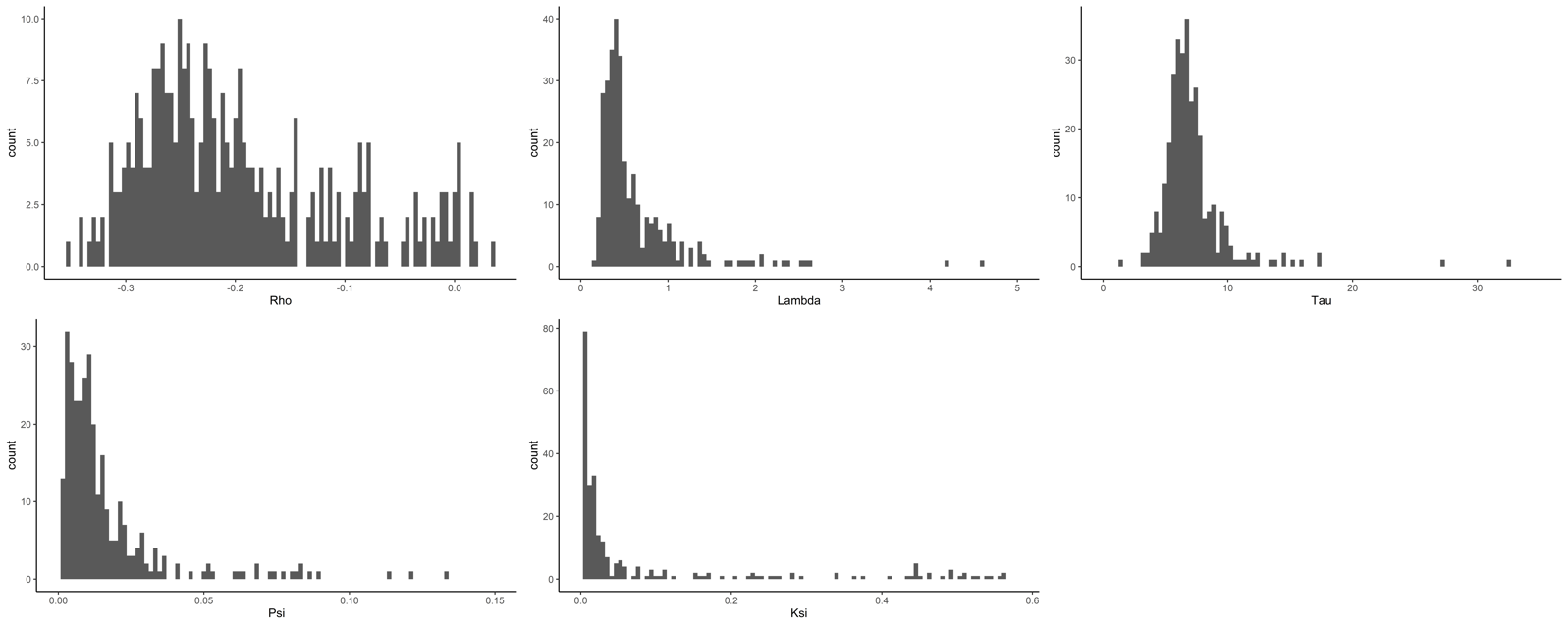


Lambda and Tau are drawn from a Phi distribution and their frequency plots above are consistent with it, even the few distant right-hand-side points.

Psi and Ksi are drawn from an Exponential Distribution and their frequency plots above are consistent with it. The only thing that stands out are that the right-hand-side tail of the Ksi plot is a little bit fatter than expected. But the number of points in that area is overall relatively small, so it is not a point of concern.

Rho is drawn from a 0.5-Phi distribution and the frequency plot is consistent with it. One small point of concern is that the right-hand-side, around 0, is fatter than expected, but this anomaly is relatively small.

# 2-Phase model Parameter Recovery

The 2-Phase model has a closed form solution (similar to the Baseline model) and therefore its parameter recovery is very good. The actual retrieved values for Probability of Burst in Exploration (Phase 1) and Exploitation (Phase 2) are as expected for this measure, and similar to the distribution of Phi, which is a similar measure in the EWMV model. The values are also reasonable with only a handful of unrealistic outliers. The Threshold parameter was not expected to follow any particular distribution.


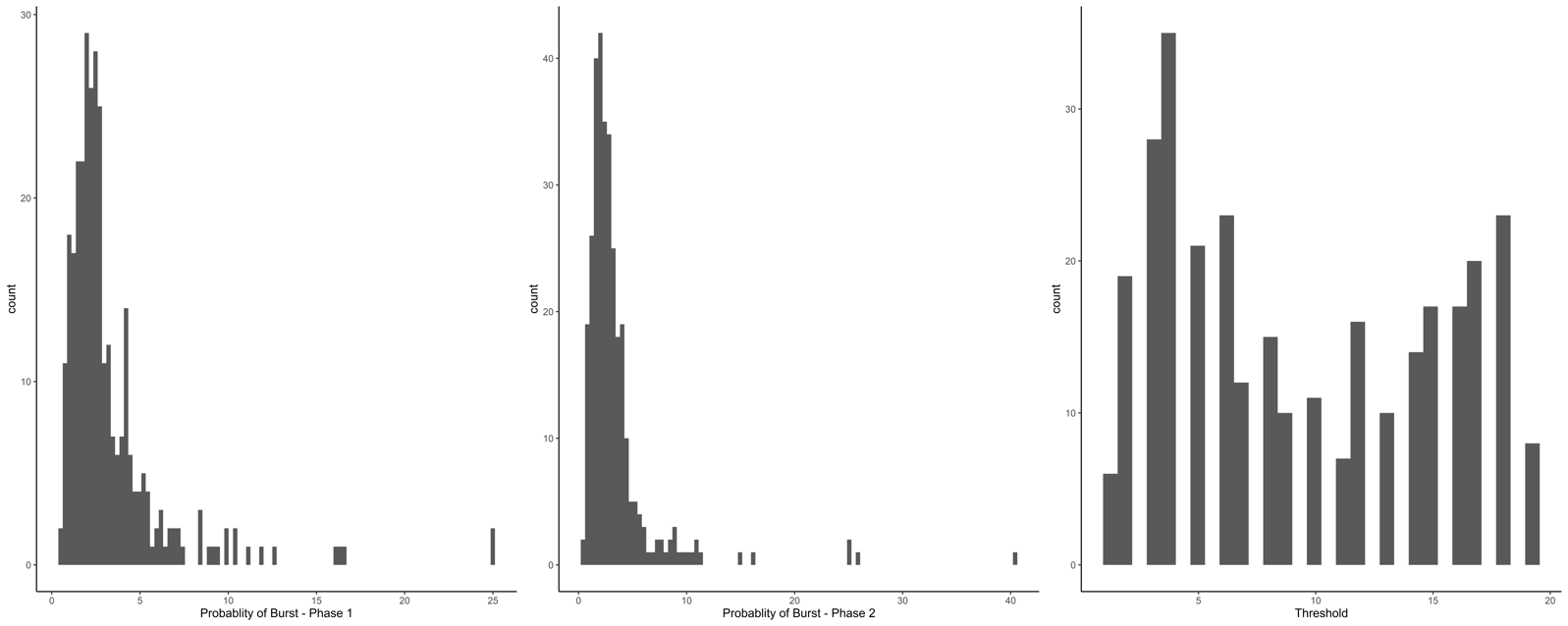


# EWMV and 2-Phase Probability Parameters Relationship

When we plot the relationship between the Probability of Burst Belief in Exploration (Phase 1) and Exploitation (Phase 2) versus the Psi parameter of the EWMV model, which is also a probability of burst belief, we see that as expected the measures of the two models have a very strong positive relationship. Also, we notice that the Probability of Burst Belief in Exploration has a stronger relationship with Psi, than the Probability of Burst Belief in Exploitation. This is expected because Psi in the EWMV model is the “prior belief of burst” and therefore it should be closer to the Probability of Burst Belief in Phase 1 than Phase 2 of the 2-Phase model.


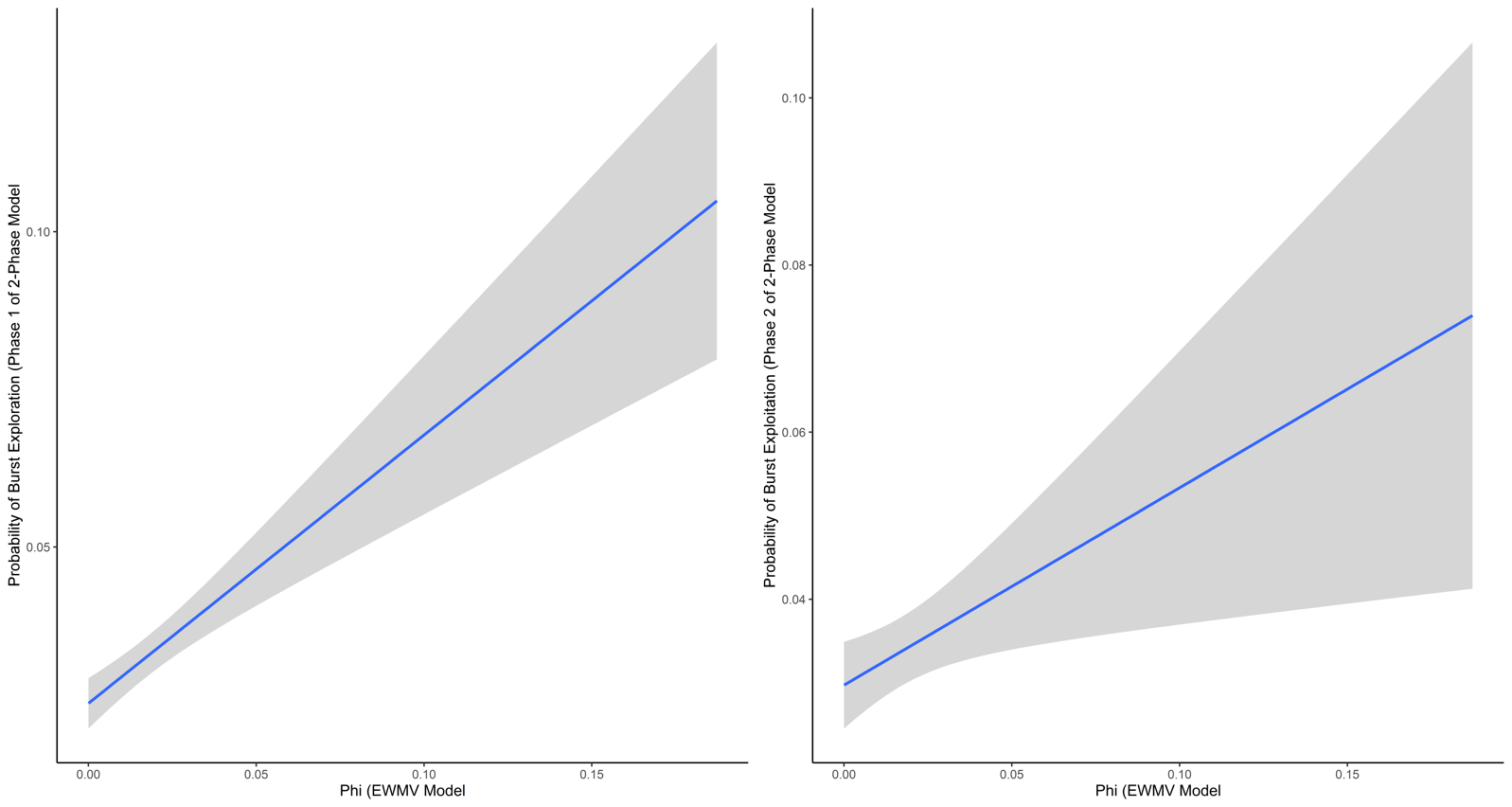


# EWMV and 2-Phase Model Simulations vs Actual

In the plots below we display the results of simulations we performed using the EWMV and 2-Phase models.

For each of the EWMV and 2-Phase models, we used each subject’s recovered parameters, and used the model formulas for evaluation and decision making to replicate their experiment behaviour in 20 trials per person. In each trial we also replicated the gradually changing probabilities per-click for the balloon to explode.

Below we display for the Actual Data, the EWMV model and the 2-Phase model:

1. the average per subject Pumps of collected trials,
2. the Pumps for each collected trial of each subject
3. the average per person explosions

For the Trial-by-Trial simulations, we found the following summary statistics:


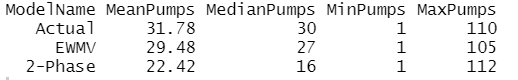


In the table below we display the summary statistics for the number of explosions in our simulations:


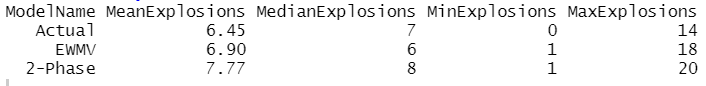


In the table above as well as in the plots below, we see that the EWMV model captures very well the distribution of the actual results. As expected, the fit of the 2-phase model was not as good as that of the EWMV model. The Actual and EWMV distributions for the Average per subject plots are very similar. In the trial-by-trial plots we see that the difference is a bit more prominent. This is because of the “irregular” preference subjects displayed for collection on “round” (at multiples of 10) number of pumps, as seen in the very high spikes on these points. Both our models captured part of this preference effect, but in a less prominent way than the actual data.

Finally, in the summary table above and the plots below, we notice that the number of explosions in the Actual and EWMV models is very similar (means of 6.45 and 6.9 respectively), while the 2-Phase model had a number of explosions a bit higher.

Therefore, we can say that in all aspects the EWMV model replicates at a high degree the Actual subject behaviour.


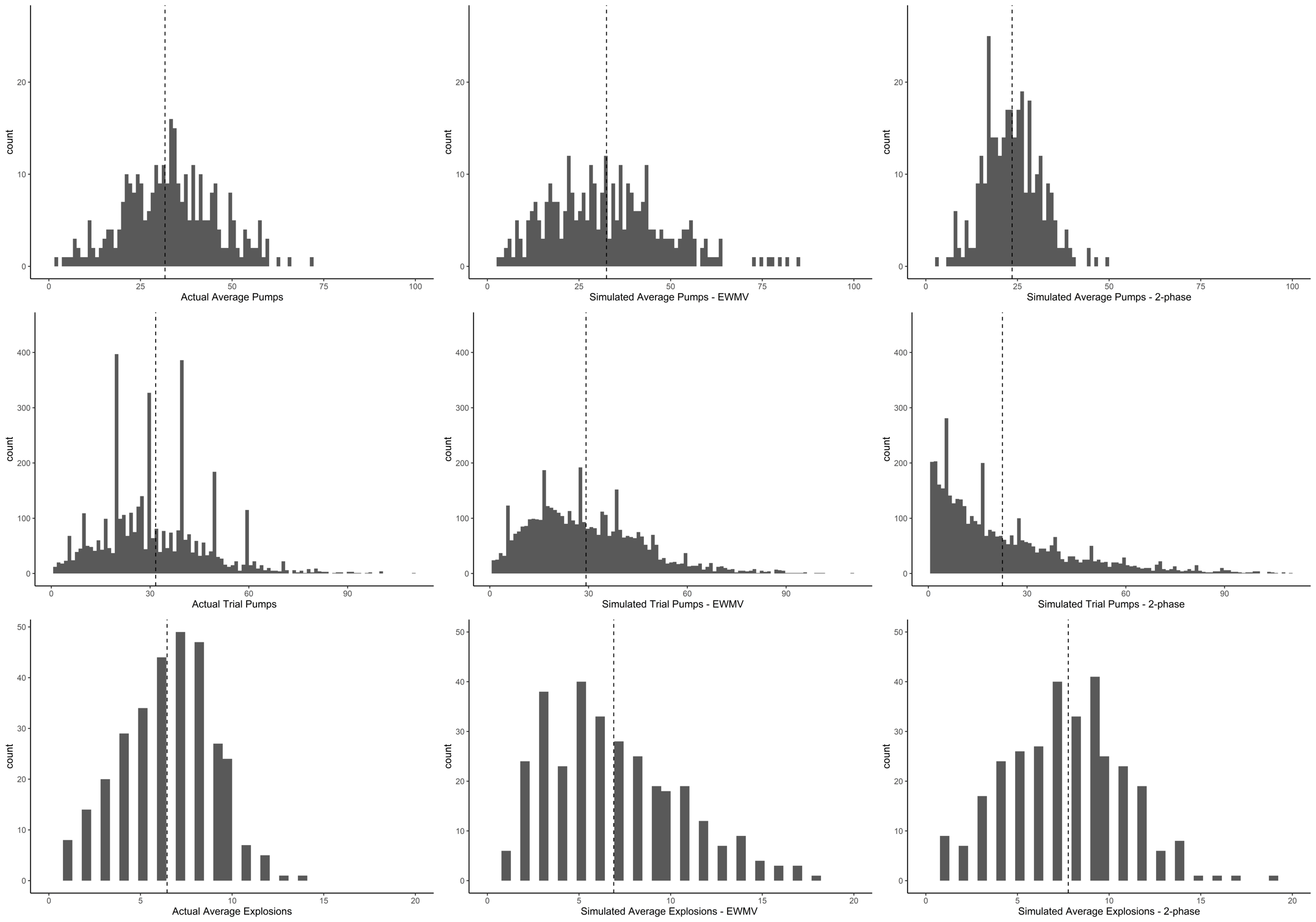

Supplement: Supplementary file 1 — Supplementary file1 (DOCX 967 kb) [file 266_2024_3910_MOESM1_ESM.docx]
